# Transcriptome profiling reveals roles of meristem regulators and polarity genes during fruit trichome development in cucumber (*Cucumis sativus* L.)

**DOI:** 10.1093/jxb/eru258

**Published:** 2014-06-24

**Authors:** Chunhua Chen, Meiling Liu, Li Jiang, Xiaofeng Liu, Jianyu Zhao, Shuangshuang Yan, Sen Yang, Huazhong Ren, Renyi Liu, Xiaolan Zhang

**Affiliations:** ^1^Department of Vegetable Sciences, Beijing Key Laboratory of Growth and Developmental Regulation for Protected Vegetable Crops, China Agricultural University, Beijing 100193, PR China; ^2^Department of Botany and Plant Sciences, University of California, Riverside, CA 92521, USA

**Keywords:** Cucumber, fruit spine, meristem regulator, polarity transcriptome, trichome.

## Abstract

Trichomes are epidermal hair-like structures that function in plant defence against biotic and abiotic stresses. Extensive studies have been performed on foliar trichomes development in *Arabidopsis* and tomato, but the molecular mechanism of fruit trichome formation remains elusive. Cucumber fruit is covered with trichomes (spines) that directly affect the appearance and quality of cucumber products. Here, we characterized the fruit spine development in wild-type (WT) cucumber and a spontaneous mutant, *tiny branched hair* (*tbh*). Our data showed that the cucumber trichome was multicellular and non-glandular, with malformed organelles and no endoreduplication. Fruit spine development was generally homogenous and marked by a rapid base expansion stage. Trichomes in the *tbh* mutant were tiny and branched, with increased density and aberrant cell shape. Transcriptome profiling indicated that meristem-related genes were highly enriched in the upregulated genes in the *tbh* versus the WT, as well as in WT spines after versus before base expansion, and that polarity regulators were greatly induced during spine base expansion. Quantitative reverse transcription PCR and *in situ* hybridization confirmed the differential expression of *CUP-SHAPED COTYLEDON3* (*CUC3*) and *SHOOT MERISTEMLESS* (*STM*) during spine development. Therefore, cucumber trichomes are morphologically different from those of *Arabidopsis* and tomato, and their development may be regulated by a distinct pathway involving meristem genes and polarity regulators.

## Summary

Cucumber fruit spine is multicellular and non-branched with no endoreduplication. Spines in the *tbh* mutant were tiny and branched. Meristem regulators and polarity genes regulate spine development in cucumber.

## Introduction

Trichomes are hair-like structures that are widely present on the surface of aerial organs of plant. As the first line of defence, trichomes function in protecting plants against insects, pathogens, and herbivores, reducing plant damage from UV irradiation, low temperature, and excessive transpiration (Hülskamp *et al.*, 1999; Hülskamp, 2004; Schellmann and Hülskamp, 2005). In addition, trichomes may help plants attract pollinators and disperse seeds (Serna and Martin, 2006). Morphologically, trichomes exhibit a broad variation in shape, size, distribution, and density, ranging from flat plates to highly ramified outgrowths, comprising a single cell or multiple cells, and being secretary glandular or non-glandular (Hülskamp *et al.*, 1999; Hülskamp, 2004; Schellmann and Hülskamp, 2005).

Extensive studies have been conducted on unicellular trichomes, especially the leaf trichomes of the model plant *Arabidopsis thaliana* (Hülskamp *et al.*, 1999; Hülskamp, 2004; Schellmann and Hülskamp, 2005; Machado *et al.*, 2009). Trichomes of *Arabidopsis* are single cells that originate from epidermis cells and are distributed on leaves, stems, and sepals in a regular pattern (Pesch and Hülskamp, 2004). During trichome morphogenesis, *Arabidopsis* leaf trichomes experience six distinct developmental stages comprising radial expansion of precursor, stalk emergence, branch formation, expansion of stalk and branches, pointed tip development, and mature trichome formation with a papillate surface (Szymanski *et al.*, 1998). Mutant analyses have identified several regulators that function in distinct developmental processes including trichome initiation and/or formation, endoreduplication, branch formation, and growth directionality (Schwab *et al.*, 2000; Szymanski *et al.*, 2000). For example, wild-type (WT) trichomes are well spaced (around three cells in between trichomes) and almost never occur next to each other (Schellmann and Hülskamp, 2005), and such patterns are regulated by an activator/inhibitor system (Schellmann *et al.*, 2002; Pesch and Hülskamp, 2004). The activators of trichome patterning consist of the WD-repeat protein TRANSPARENT TESTA GLABRA1 (TTG1) (Galway *et al.*, 1994; Walker *et al.*, 1999), the basic helix–loop–helix (bHLH) protein GLABRA3 (GL3) or ENHANCER OF GLABRA3 (EGL3) (Payne *et al.*, 2000; Zhang *et al.*, 2003), and two R2-R3 type-MYB transcription factors, GLABRA1 (GL1) and MYB23 (Oppenheimer *et al.*, 1991), whereas the single-repeat MYB proteins TRIPTYCHON (TRY) (Schellmann S *et al.*, 2002; Pesch and Hülskamp, 2011), CAPRICE (CPC) (Wada *et al.*, 1997, 2002), ENHANCER OF TRY AND CPC1 (ETC1), ETC2, and CAPRICE-LIKE MYB3 (Esch *et al.*, 2004; Kirik *et al*., 2004*a*, *b*; Simon *et al.*, 2007) act partially redundantly as negative regulators. After gaining the trichome cell fate, the incipient trichome cells switch from mitotic divisions to endoreduplication, reaching a DNA content of 32C (Hülskamp *et al.*, 1994; Schellmann and Hülskamp, 2005), and this cell-cycle control is co-ordinately regulated by many factors including SIAMESE (SIM), a nuclear-localized plant-type cell-cycle regulator that represses endoreduplication cycles, and the plant cytohormone gibberellin signalling regulator SPINDLY (SPY), which promotes endoreduplication (Chien and Sussex, 1996; Jacobsen *et al.*, 1996; Churchman *et al.*, 2006). Furthermore, trichome DNA content resulting from endoreduplication cycles positively correlates with branch numbers. WT trichomes on rosette leaves usually have three to four branches, while mutants with increased DNA content such as *try* and *spy* have trichomes with up to eight branches (Hülskamp M *et al*., 1994; Perazza *et al.*, 1999).

Compared with unicellular trichomes, the development and regulatory networks of multicellular trichomes in plants are much less understood, with most of the work being carried out in tomato. Multicellular trichomes in tomato are classified into I–VII types and, based on whether trichomes contain or secret a mixture of chemicals that confer resistance against insects or pathogens, they are classified into glandular (types I, IV, VI, and VII) or non-glandular (types II, III, and V) trichomes (Luckwill, 1943; Kang *et al.*, 2010). The majority of trichome research in tomato has focused on the morphology, chemical composition, defence against herbivores, and developmental regulators of glandular trichomes, and it has been shown that there are both conserved and divergent regulatory pathways involved in different types of trichomes (Li *et al.*, 2004; Kang *et al.*, 2010; Yang *et al.*, 2011; Deng *et al.*, 2012; Tian *et al.*, 2012). So far, little is known about the developmental process of multicellular non-glandular trichomes and the underlying regulatory molecular mechanisms.

Cucumber (*Cucumis sativus* L.) is one of the most important vegetable crops and has been cultivated worldwide for over 3000 years (Huang *et al.*, 2009). Cucumber fruit are usually harvested at 1–2 weeks after anthesis and then used as fresh product or processed into pickles. The development of cucumber fruit follows a stereotypical pattern with visible external and internal morphological changes (Ando *et al.*, 2012). During early fruit development, deep ridges along the length of the fruit cover the surface of the fruit, and densely spaced fruit spines (trichomes on cucumber fruits) are randomly scattered relative to the ridges (Ando *et al.*, 2012). The fruit spine is of agricultural importance in affecting the appearance and flavour of cucumber products. To the best of our knowledge, there are no reports about the regulation of fruit spine development, and there is no detailed characterization of any cucumber mutant with disturbed trichome development. In this study, we compared and explored the developmental process and internal cell structures of cucumber trichomes in the WT and in a glabrous mutant, *tiny branched hair* (*tbh*). We further performed comparative transcriptome profiling analyses to identify genes and gene networks that may be involved in cucumber trichome development. We found that cucumber trichomes are multicellular and non-glandular, with no branches or endoreduplication. Development of fruit spines was generally homogenous and marked by a rapid spine base expansion stage. Furthermore, we found that meristem-related genes and polarity regulators participate in the development of fruit spine in cucumber.

## Materials and methods

### Plant materials

Cucumber inbred line R1407 (WT) and *tbh* mutant plants were grown at two generations every year in a greenhouse in the experimental field of the China Agricultural University in Beijing. Pest control and water control were carried out according to standard protocols.

### Scanning electron microscopy (SEM)

SEM of cucumber trichomes was performed on young fruits with eight different lengths (0.5, 1.0, 1.6, 1.85, 2.3, 3.5, 4.3, and 6.5cm) and two stages of leaves (juvenile and mature). Samples were fixed with 2.5% glutaraldehyde at 4 °C for approximately 24h, washed with PBS (pH 7.2) three times and post-fixed in 1% (v/v) OsO_4_. The samples were then dehydrated through an ethanol series (30, 50, 70, 80, 90, and 100%, three times), critical-point dried using a desiccator (HCP-2; Hitachi), and coated with gold palladium (EIKO IB-3). Images were taken with a Hitachi S-4700 scanning electron microscope using a 2kV accelerating voltage.

### Transmission electron microscopy (TEM)

Fruit spines were isolated from WT cucumber fruits of 1.6–1.8cm in length using fine tweezers under a dissecting microscope. Spines and leaves were fixed in 2.5% (w/v) glutaraldehyde and rinsed thoroughly with 0.1M phosphate buffer. Samples were post-fixed with 1% Hungry acid, washed in 0.1M phosphate buffer, dehydrated through an acetone series (30, 50, 70, 80, 90, and 100%), and then embedded in Spurr’s resin. Thin sections were cut with a LEICA UC6I microtome and examined with a JEM-123O scanning transmission electron microscope.

### Flow cytometry analysis

Flow cytometry was performed as described previously (Galbraith *et al.*, 2001) using a BD FACSCalibur analyser. Spines from 12 WT cucumber fruit of 1.6–1.8cm in length were isolated and pooled as a sample. To minimize contamination from trichome tissues, leaves from *tbh* mutant plants were used as a negative control. Nuclei from spines or leaves were prepared and stained with 4’,6-diamidino-2-phenylindole (DAPI) as described previously (Galbraith *et al.*, 2001). Histograms of DAPI fluorescence indicated the relative DNA content in each sample.

### Plant materials for digital gene expression (DGE) analysis

Two sets of transcriptome profiling experiments were carried out using the DGE approach. Samples were collected at the same time on the same day. The first set was to compare the transcriptome profiles of pericarps (epidermis plus trichome) from cucumber fruit in WT and the *tbh* mutant. Pericarps of around 0.2cm thick were peeled off from 1.6–1.8cm cucumber fruits. Pericarps of three fruits from different plants were pooled together as one biological sample for each genotype. The second set was used to compare spine-specific transcriptome profiles in two developmental stages. Fruit spines from WT cucumber fruit that were 1.6 or 0.5cm in length were isolated under a dissecting microscope, and spines from at least five fruits from different plants were pooled as one biological sample. Two biological replications were performed for each set of experiments.

### DGE library construction and sequencing

DGE library construction was performed as described previously (Eveland *et al.*, 2010), with minor modifications. Briefly, total RNA was extracted using a Huayueyang RNA extraction kit (Huayueyang, China), and 6 µg of total RNA was used for constructing each library. Double-stranded cDNAs were synthesized using oligo(dT) beads (Invitrogen). The cDNAs were then digested with an anchoring restriction enzyme (*Nla*III) and ligated to a 5’ adapter, which contains a recognition site for the type IIS restriction enzyme *Mme*I (New England Biolabs). Following *Mme*I digestion, a 3’ adapter was ligated. Tags flanked by both adapters were enriched by PCR for 15 cycles. The PCR products were run on a 6% polyacrylamide gel, and the ~105bp band was excised and purified. The DNA samples were sequenced on an Illumina HiSeq 2000 sequencer for 49 cycles.

### Bioinformatics analysis of DGE data

Raw reads were trimmed for the adapter sequence and only 21 nt clean tags with a count of at least 2 in a library and without any ambiguous nucleotide (‘N’) were retained for further analysis. Clean tags were clustered into unique tags, which were mapped to the cucumber genome sequence (http://cucumber.genomics.org.cn, v.2i) (Huang *et al.*, 2009) using the SOAP2 software (Li *et al.*, 2009), allowing up to one mismatch. To recover tags that cover intron–exon junctions, unmapped tags were aligned to the annotated cucumber transcripts using SOAP2, allowing up to one mismatch. If a tag was mapped to multiple locations in the genome, the tag count was divided by the number of mapped locations. The expression value of a gene was calculated as the total count of all tags that were mapped to the sense strand of the gene region. Following the convention set as described (Eveland *et al.*, 2010), gene boundaries were extended for 300bp on both 5’ and 3’ ends to maximize the capture of complete untranslated regions. EdgeR (Robinson *et al.*, 2010) was used to identify genes that were differentially expressed in fruit spines of WT versus *tbh* mutant, and fruit spines from cucumber fruit of 0.5 versus 1.6cm long. The method of Benjamini and Hochberg (1995) was used for adjustment for multiple comparisons. A false discovery rate (FDR) of 0.05 was used as the significance cut-off.

Sequencing data were deposited in the Gene Expression Omnibus (GEO) database at the National Center for Biotechnology Information under accession number GSE49607.

### Gene Ontology (GO) term enrichment analysis

Because the GO terms are not well annotated for cucumber genes, we used the best homologues in *Arabidopsis* for GO term enrichment analysis. We first used the cucumber annotated proteins to search the *Arabidopsis* proteins (TAIR10) using BLASTP with an e-value cut-off of 1e–5, and identified the best homologue (with the lowest e-value) in *Arabidopsis* for each cucumber gene. For the genes that were upregulated and downregulated, respectively, in each of the two pairwise transcriptome comparisons, we collected the corresponding *Arabidopsis* best homologues and used the GOEAST software (Zheng and Wang, 2008) to test for GO term enrichment. GOEAST was run with default parameters except for the use of algorithms to eliminate local dependencies between GO terms (Alexa *et al.*, 2006).

### Quantitative and semi-quantitative reverse transcription (RT)-PCR

To confirm the results of the DGE analyses, samples for RT-PCR were independently collected from pericarps or spines from the same batch of plants at the same time as in the DGE analyses. For Supplementary Table S4 (available at *JXB* online), RNA samples were exacted from the third true leaves or roots of 4-week-old cucumber seedlings. Total RNA was isolated with a Huayueyang RNA extraction kit and then reverse transcribed by Moloney murine leukemia virus reverse transcriptase using random primers. Quantitative RT-PCR (qRT-PCR)was performed on an Applied Biosystems 7500 real-time PCR system using SYBR Premix Ex *Taq* (TaKaRa). Both qRT-PCR and semi-quantitative PCR were repeated with three biological samples. The cucumber *UBIQUITIN* gene was used as reference control to normalize the expression data (Wan *et al.*, 2010). The gene-specific primers are listed in Supplementary Table S5 available at *JXB* online.

### *In situ* hybridization

Cucumber young fruits from WT and *tbh* mutant were fixed and hybridized as described previously (Zhang *et al.*, 2013). *In situ* probes were generated through PCR amplification using gene-specific primers with T7 and SP6 RNA polymerase-binding sites. The gene-specific primers are listed in Supplementary Table S5 available at *JXB* online.

## Results

### Morphological characterization of the *tbh* mutant in cucumber

**Fig. 1.**
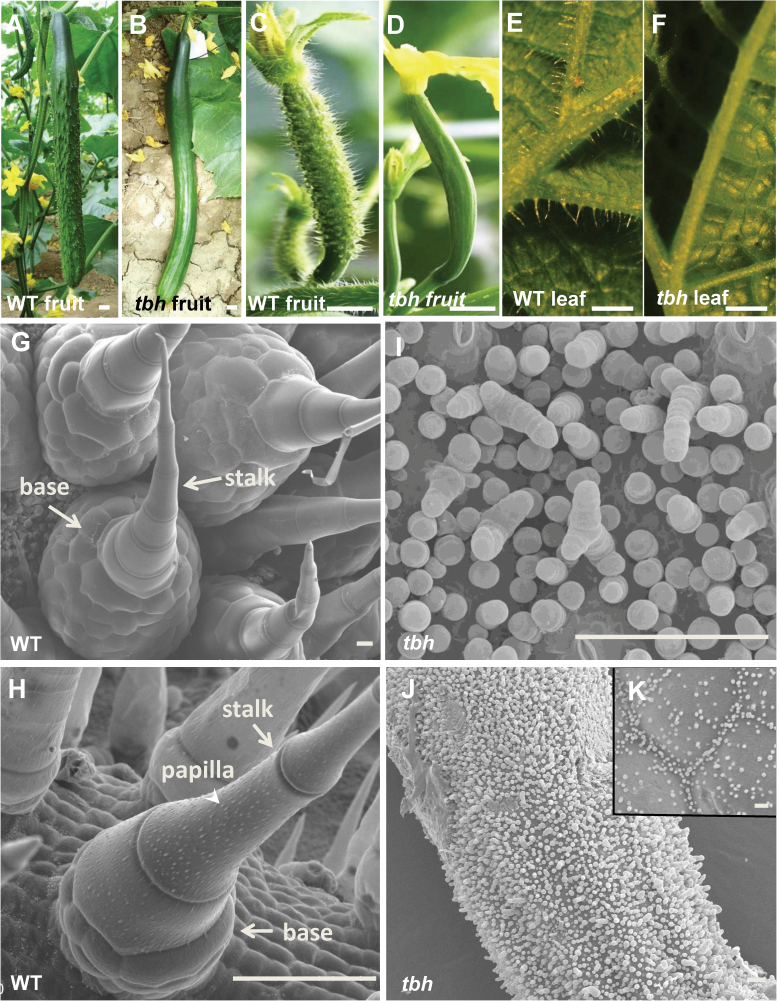
Morphological characterization of the *tbh* mutant. (A, B) Commercially mature fruit of WT (A) and *tbh* mutant (B). (C–F) Light microscope images of young cucumber fruit around anthesis (C, D) and the back of leaves (E, F) in WT (C, E) and *tbh* mutant (D, F). (G–K) SEM images of fruit spines (G, I, J) and leaf trichomes (H, K) in WT (G, H) and *tbh* mutant (I–K). Bars, 1cm (A–F); 50 µm (G–K). (This figure is available in colour at *JXB* online.)

From the cucumber inbred line R1407, a spontaneous mutant, *tbh*, was identified by its ‘glabrous’ phenotype: hairless foliage and smooth fruit surface. We further stabilized the *tbh* mutation via six generations of selfing prior to this study. By the commercially mature stage, the WT fruit was dark green with spines and warts that were evenly distributed on the fruit surface (Fig. 1A and Supplementary Fig. S1A available at *JXB* online), whereas the *tbh* fruit appeared to be smooth and shining, with no noticeable spines or warts (Fig. 1B). During the stage of anthesis, WT young fruit was covered with densely spaced spines (Fig. 1C), while *tbh* fruit was glabrous (Fig. 1D). We next examined the cucumber trichomes with an optical microscope (Fig. 1E, F, Supplementary Fig. S1B–G available at *JXB* online). In the WT plants, leaves (Fig. 1E), tendrils (Supplementary Fig. S1B available at *JXB* online), stems (Supplementary Fig. S1C available at *JXB* online), and male flower buds (Supplementary Fig. S1D available at *JXB* online) were all covered with hairs, but in the *tbh* mutant plants, no evident trichomes were observed throughout the plant (Fig. 1F, Supplementary Fig. S1E–G available at *JXB* online). To characterize the morphology of cucumber trichomes in details, we observed fruit spines and leaf trichomes with SEM. Unlike the single-cell branched trichomes in *Arabidopsis* or multiple types of trichomes in tomato (Hülskamp *et al.*, 1994; Kang *et al.*, 2010; Tominaga-Wada *et al.*, 2013), there were only two types of trichomes in cucumber, and both were multicellular (Supplementary Fig. S2A available at *JXB* online). Type I trichomes were tiny with a three-to-five-cell base topped with a four-to-eight-cell head (Supplementary Fig. S2B available at *JXB* online). Type I trichomes were shown to be involved in cuticle formation (Samuels *et al.*, 1993). Type II trichomes were much bigger, with a conical shape, and were non-glandular and branchless (Fig. 1G, H). Type II trichomes were the dominant type that we observed in cucumber, and thus will be our focus hereafter. Each mature type II trichome was comprised of a base and a stalk. The base was made of hundreds of spherical-shaped cells whose sizes were smaller than those of the stalk cells. The stalk was consisted of three to seven cylindrical-shaped cells plus a pyramid-shaped apical cell (Fig. 1G, H). Therefore, the cucumber trichomes appeared sharp on the top. The leaf trichomes and fruit spines in cucumber had a similar structure and morphology, but there were three differences: (i) the size of the spine base was much bigger than that in the leaf trichome; (ii) white papilla (arrowhead in Fig. 1H) was observed on mature leaf trichomes but not on fruit spines (compare Fig. 1G and H); and (iii) trichome spacing was generally even on the fruit but uneven on cucumber leaves. There were more trichomes on the veins than other leaf areas (Fig. 1E and Supplementary Fig. S1A available at *JXB* online). In the *tbh* mutant, trichome morphology was strikingly defective. The most obvious characteristics of *tbh* trichomes were that they were ‘tiny’ with a greatly reduced number of cells, and that the cell shape and organization were so aberrant that it was difficult to separate the base and the stalk (Fig. 1I, K). Moreover, trichomes in the *tbh* mutant varied from no branches to up to four branches, and the apical cell was spherical (Fig. 1I). Hence, the top of the trichome in the *tbh* mutant was round, which was dramatically different from the ‘sharp tip’ in the WT. Furthermore, the density of trichome distribution in the *tbh* mutant was much higher compared with that in the WT, varying from 4-fold higher in the young leaves to around 118-fold more trichomes on the fruit of *tbh* during anthesis compared with those in WT, suggesting that TBH functions in both trichome outgrowth and trichome spacing. In addition, there was no noticeable difference between the leaf trichome and fruit spine in the *tbh* mutant with regard to trichome structure and morphology, despite the distribution pattern being different [distributed evenly on the fruit surface (Fig. 1J) but unevenly on the leaf surface (Fig. 1K)]. Since fruit spines directly affect the appearance and quality of the cucumber fruit, the rest of our study focused on the characterization of fruit spines.

### Internal cell structure of cucumber trichomes

**Fig. 2.**
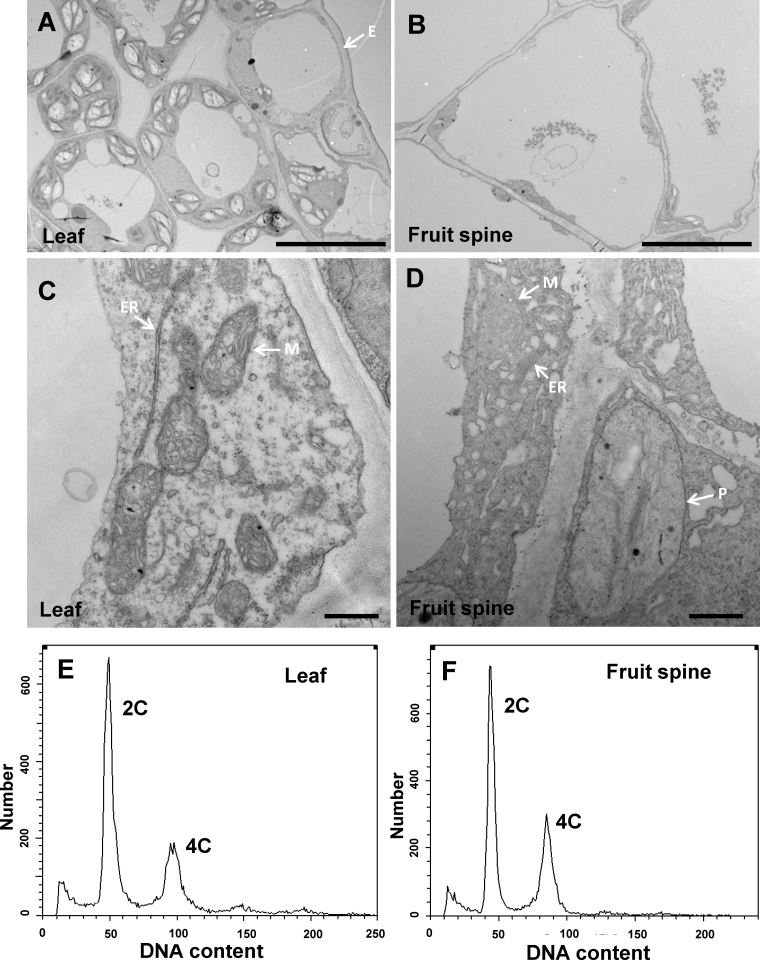
Internal structure of cucumber trichome cells. (A–D) Transmission electron micrographs of the abaxial leaf cells (A, C) or the base cells of fruit spine (B, D) from WT cucumber. Bars, 10 µm (A, B); 500nm (C, D). (E, F) Flow cytometric analysis of the DNA content of nuclei in the *tbh* mutant leaf tissue (E) and WT fruit spines (F). The histograms show the DNA content of each nucleus. E, epidermal cell; P, plastid; ER, endoplasmic reticulum; M, mitochondria.

The unicellular *Arabidopsis* trichomes have been thought to originate from the epidermal cells (Marks, 1997; Hülskamp *et al.*, 1999; Larkin *et al.*, 2003), but comparison of the internal structure of trichomes and epidermal cells is lacking due to technical difficulties of fixing individual trichome cells. Taking advantage of the multicellular property of cucumber trichomes and the large base in fruit spines, we explored the cellular structure of cucumber trichomes using TEM (Fig. 2A–D). Compared with the abaxial leaf epidermal cells (Fig. 2A, C), the base cells of fruit spine were much larger, with an enormous central vacuole and the internal organelles being squeezed close to the cell wall (Fig. 2B). The endoplasmic reticulum and mitochondria appeared to be shorter and swollen in the cucumber trichome cells, probably due to the presence of a large central vacuole (Fig. 2D). Furthermore, misshaped plastids were often observed in the spine base cells but not in the leaf epidermal cells. The light green colour of the cucumber spine base supported the existence of coloured plastids or even chloroplasts in the spine cells (Supplementary Fig. S1A available at *JXB* online). The striking differences in morphology and cellular structure between cucumber and *Arabidopsis* trichomes suggest that cucumber trichomes may have a distinct origin from the epidermal-derived *Arabidopsis* trichomes and may have a divergent developmental mechanism.

Previous studies have shown that the nuclear DNA content, an indicator of endoreduplication, is often positively correlated with cell size (Melaragno *et al.*, 1993), and that *Arabidopsis* trichome cells undergo four rounds of endoreduplication resulting in a genomic DNA content of 32C (Hülskamp *et al.*, 1994). To determine whether endoreduplication contributed to the enlarged cucumber trichome cells, we measured the DNA content of mature fruit spines using flow cytometry. Surprisingly, no difference in nuclear ploidy levels was observed between fruit spines and leaf tissue (2C and 4C) (Fig. 2E, F), suggesting that cucumber trichome cells did not undergo any endoreduplication.

### Developmental stages of cucumber fruit trichomes

**Fig. 3.**
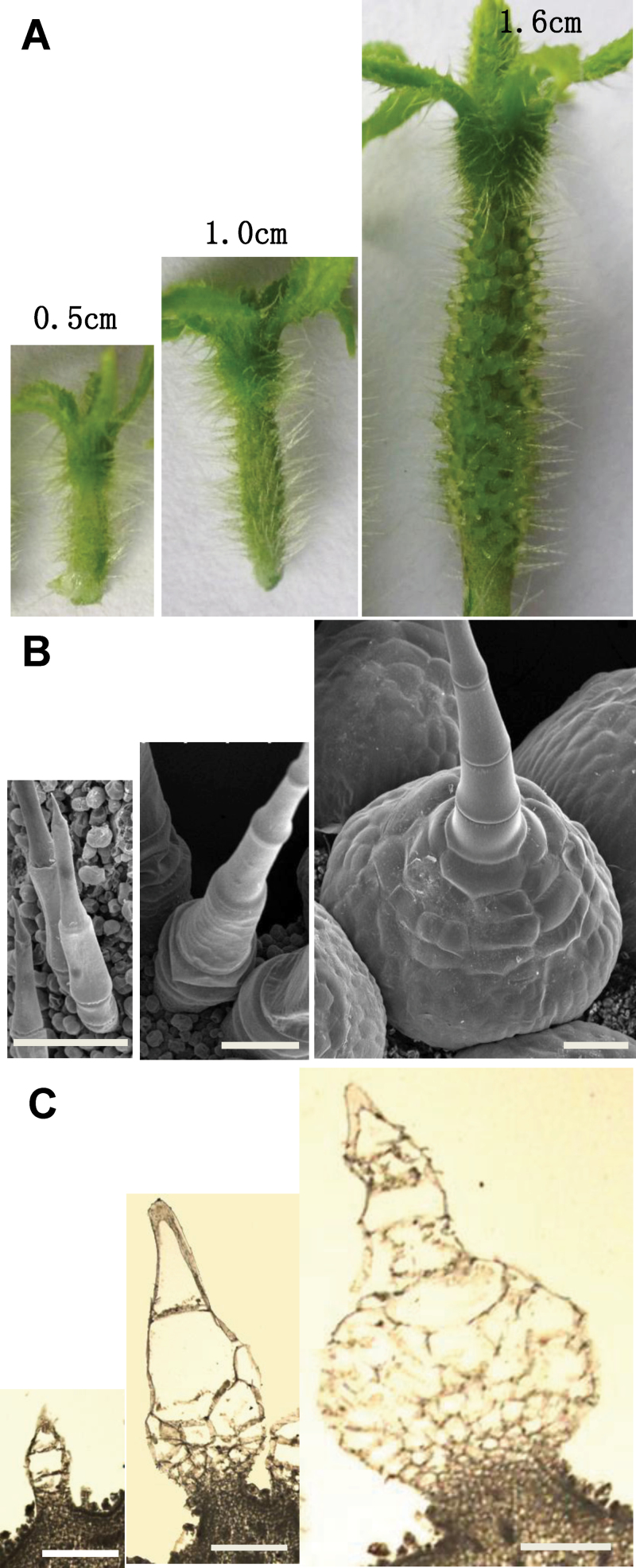
Key developmental stages of spine base expansion in cucumber fruits. Digital camera images (A), SEM images (B) and paraffin sections (C) of cucumber spine before base expansion (left, fruit length around 0.5cm), during base expansion (middle, fruit length around 1.0cm), and after rapid base expansion (right, fruit length around 1.6cm). Bars, 200 µm (B, C).). (This figure is available in colour at *JXB* online.)

To characterize further the developmental process of cucumber trichomes in detail, we observed the spines of eight developmental stages of WT cucumber fruit (based on fruit size) using SEM (Supplementary Fig. S3 available at *JXB* online). Unlike leaf trichomes, which usually undergo distinct stages at the same time, even on the same leaf, cucumber fruit spines were generally homogenous (at the same developmental stage) on the same fruit (Supplementary Fig. S3 available at *JXB* online). The most dramatic change during spine development was the expansion of the spine base. When the fruit length was about 0.5cm long (approximately 7 d before anthesis), the spine consisted of a few cells that were organized in one tile, with a ‘sharp’ stalk on the top and a single-cell base (Fig. 3, left). The spine base experienced a rapid expansion through cell division when the fruit length was between 0.5 and 1.5cm (Fig. 3, middle). When the fruit length reached around 1.6cm (approximately 4 d before anthesis), the spine base became a dome-shaped body that contained hundreds of cells and could be easily observed by bare eyes (Fig. 3, right). The spine base continued to expand until the fruit reached around 4.3cm (during anthesis), after which its shape and size were maintained as relatively constant (Supplementary Fig. S3 available at *JXB* online). Based on these observations, we defined a fruit length of 0.5–1.6cm as the key stage for spine base expansion in our cucumber cultivar.

### Transcriptome analyses of cucumber fruit spines

**Fig. 4.**
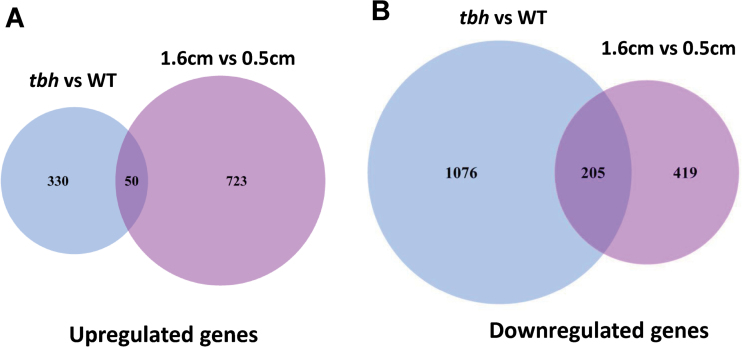
Venn diagrams of differentially expressed genes that were significantly upregulated (A) or downregulated (B) (FDR <0.05) in the *tbh* mutant (blue) or in the spines of 1.6cm fruit (purple). (This figure is available in colour at *JXB* online.)

To identify genes and gene networks that were involved in the multicellular fruit spine development in cucumber, we performed two sets of genome-wide expression analyses using the DGE approach (Eveland *et al.*, 2010). One set was to compare the transcriptome profiles of the pericarps (epidermis plus spines) of 1.6–1.8cm cucumber fruits between WT and the *tbh* mutant, and the other set was to examine WT spine-specific expression profiles before (fruit length around 0.5cm) versus after (fruit length around 1.6cm) spine base expansion. Two biological replicates were performed for each set, and thus eight DGE libraries were sequenced (two comparisons×two tissues×tworeplicates). We generated 6.95–7.49 million raw reads from each library. After adapter sequence and low-quality tags had been removed, we obtained 6.80–7.33 million clean tags that were 21 nt long and with counts of at least 2 in a library (Supplementary Table S1 available at *JXB* online). We clustered clean tags into unique tags, which were mapped to the cucumber genome and annotated transcripts. We summarized the total counts of tags that were mapped to the sense strand of each annotated cucumber gene region, which represented the expression levels in each sample. Using an FDR of 0.05 as the significance cut-off, we found that the number of downregulated genes (1281) was dramatically more than that of upregulated genes (380) in the *tbh* mutant compared with the WT (Fig. 4, Supplementary Table S2 available at *JXB* online), suggesting that the *TBH* locus may function as a major activator during fruit spine development. In the 2 (1.6 vs 0.5cm) of transcriptome comparison, we also found that 1397 genes were differentially expressed, in which 773 genes were upregulated and 624 genes were downregulated in the spines of 1.6cm long cucumber fruit compared with the spines of 0.5cm fruit (Fig. 4, Supplementary Table S3 available at *JXB* online).

To validate the differentially expressed genes (DEGs) identified by DGE, we performed qRT-PCR assays using independently generated cucumber pericarp and fruit spine samples during the same developmental stages as those used in DGE. We randomly chose 20 DEGs for qRT-PCR analysis, in which eight were from set 1 (*tbh* vs WT) and 12 were from set 2 (1.6 vs 0.5cm). The qRT-PCR and DGE data showed close agreement (Pearson correlation coefficient 0.996, *P*=1.59E–21) (Table 1), indicating that the DGE results were highly reliable.

**Table 1.** qRT-PCR confirmation of differentially expressed cucumber genes identified by DGE

Gene ID	Putative annotation	DGE *P* value	DGE fold change	qRT-PCR fold change
***tbh* vs WT**				
Csa1G064670	ATML1 (MERISTEM LAYER 1)	1.6E–05	3.2	3.2±0.41
Csa6G514870	PDF2 (PROTODERMAL FACTOR 2)	1.5E–05	2.3	2.2±0.09
Csa4G256430	BOP2 (BLADE ON PETIOLE2)	3.0E–03	2.7	4.1±0.57
Csa6G355380	CUC3 (CUP-SHAPED COTYLEDON3)	6.4E–39	–1638.5	–481.4±1.59
Csa2G167190	BMY7 (BETA-AMYLASE 7)	7.9E–13	–15.8	–5.4±0.43
Csa1G628000	LRP1 (LATERAL ROOT PRIMORDIUM 1)	7.5E–20	–6.5	–7.5±0.37
Csa6G501990	HB-5 (HOMEOBOX PROTEIN 5)	2.4E–51	–142.9	–5.8±1.78
Csa3G824850	MYB106 (MYB DOMAIN PROTEIN 106)	3.3E–19	–12.7	–20.6±1.19
**1.6cm vs 0.5 cm**				
Csa7G041370	STM (SHOOT MERISTEMLESS)	3.4E–09	37.8	22.8±0.89
Csa6G012240	LBD21 (LOB DOMAIN-containing PROTEIN 21)	5.2E–07	10.4	12.6±0.94
Csa5G172270	GA5(GA REQUIRING 5)	1.3E–05	8.4	3.4±0.44
Csa3G895630	ATHB-8 (HOMEOBOX GENE 8)	4.5E–07	6.3	2.1±0.75
Csa6G496390	ANT (AINTEGUMENTA)	1.2E–04	4.0	3.7±0.71
Csa6G426940	YAB2 (YABBY2)	6.7E–04	3.7	3.0±0.88
Csa2G348930	KNAT6 (KNOTTED1-LIKE HOMEOBOX GENE 6)	2.0E–03	3.6	3.1±0.61
Csa3G194380	KAN1 (KANADI1)	1.8E–03	3.0	4.5±0.65
Csa3G143580	SHY (SHORT HYPOCOTYL)	1.4E–05	–5.1	–7.4±0.63
Csa1G064670	ATML1 (MERISTEM LAYER 1)	2.2E–05	–6.4	–11.2±0.63
Csa1G397130	SHY2 (SHORT HYPOCOTYL 2)	1.6E–06	–7.1	–3.7±0.79
Csa6G367090	AP1 (APETALA1)	1.2E–04	–7.2	–4.7±1.18

### Meristem regulators and transcription factors implicated in cucumber fruit spine development

**Fig. 5.**
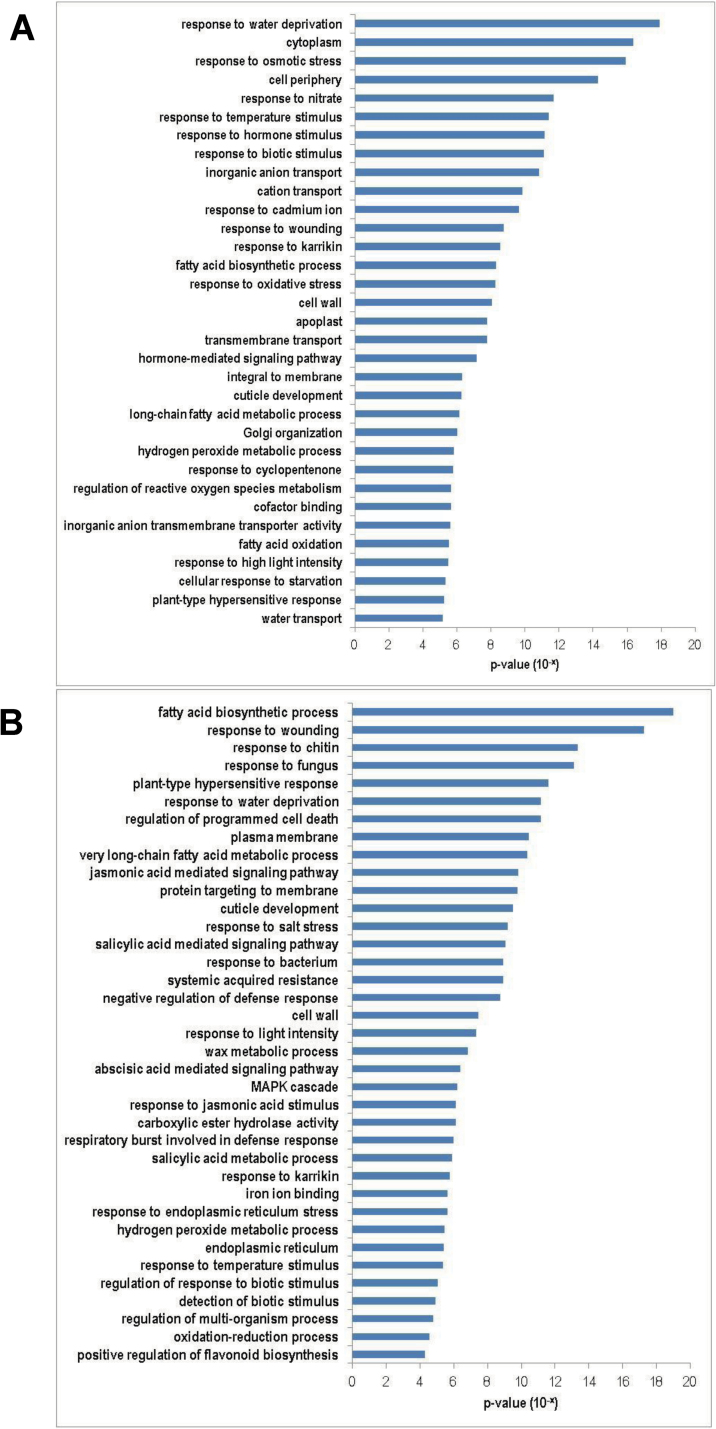
GO terms that were significantly enriched (*P*<0.01) in the downregulated genes in the *tbh* mutant (A) or in the spines of 1.6cm long fruit (B). GO terms were sorted based on *P* values.

**Fig. 6.**
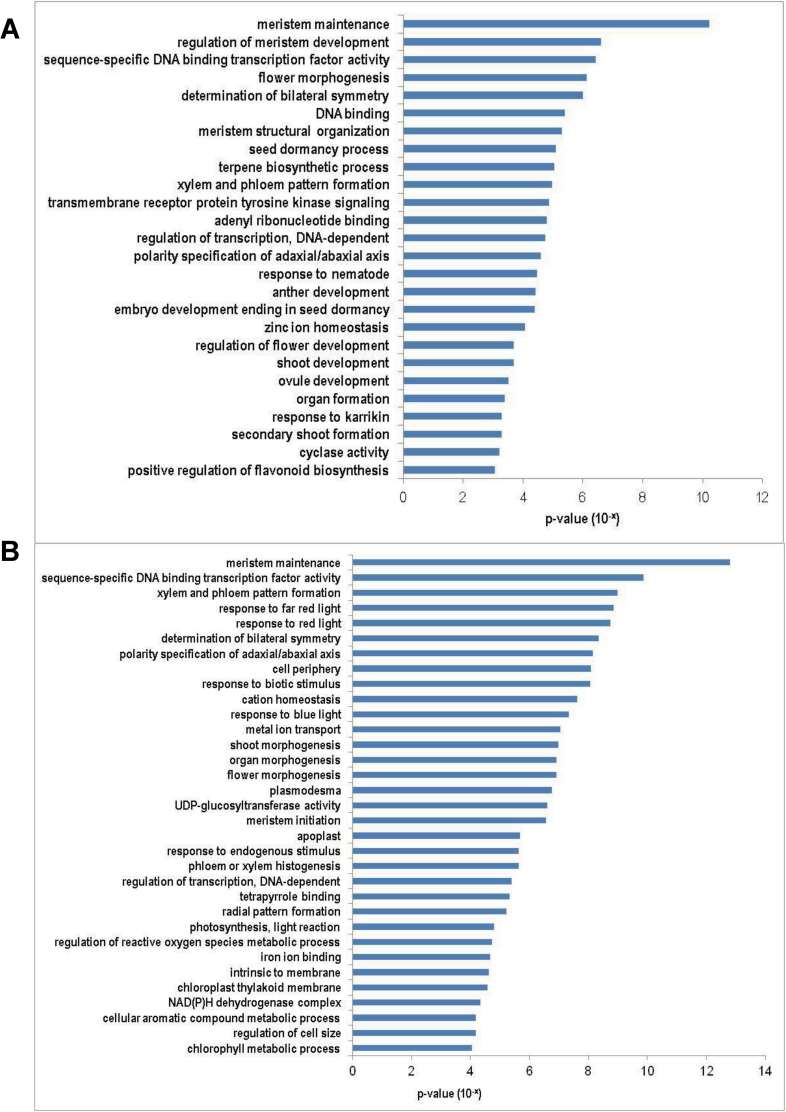
GO terms that were significantly enriched (*P*<0.01) in the upregulated genes in the *tbh* mutant (A) or in the spines of 1.6cm fruit (B). GO terms were sorted based on *P* values.

To analyse the functions of DEGs identified by DGE, GO term enrichment analyses were carried out for the up- and downregulated genes in both sets of data. Stress-related and metabolic pathway genes were significantly enriched in the DEGs that were downregulated in both *tbh* mutant (Fig. 5A) and spines of 1.6cm fruit (after base expansion) (Fig. 5B). For example, GO terms ‘response to water deprivation’ (*P*=1.2E–18), ‘cytoplasm’ (*P*=4.1E–17), and ‘response to osmatic stress’ (*P*=1.7E–16) were the top three significantly enriched groups in genes that were downregulated in the *tbh* mutant (Fig. 5A), whereas ‘fatty acid biosynthetic process’ (*P*=1.0E–19), ‘response to wounding’ (*P*=5.6E–18), and ‘response to chitin’ (*P*=4.4E–14) were the most significantly enriched GO terms in the downregulated genes in the spines of 1.6cm fruit. In contrast, meristem regulators and transcription factors were significantly enriched in the genes that were upregulated in both the *tbh* mutant (Fig. 6A) and the spines of 1.6cm fruit (after base expansion) (Fig. 6B, Tables 2 and 3). The most significantly enriched GO term was ‘meristem maintenance’ for both sets of data, with a *P* value of 6.12E–11 and 1.56E–13 in the *tbh* vs WT and the 1.6 versus 0.5cm comparisons, respectively. Accordingly, many well-known meristem regulators were significantly induced in the *tbh* mutant (Table 2) and in the spines of 1.6cm fruit (Table 3). For example, the expression levels of *REPLUMLESS* (*RPL*) and *MERISTEM LAYER* 1(*ATML1*) were 3.6- and 3.2-fold higher, respectively, in the *tbh* mutant compared with those in the WT (Table 2), and the expression of *SHOOT MERISTEMLESS* (*STM*) and *ZWILLE* (*ZLL*) increased 37.8- and 9.0-fold, respectively, in the spines of 1.6cm fruit compared with those in 0.5cm fruit (Table 3). The GO term ‘sequence-specific DNA-binding transcription factor activity’ was also significantly enriched in the genes that were upregulated in both *tbh* mutant (*P*=3.67E–07) and the spines of 1.6cm fruit (*P*=1.34E–10), suggesting that meristem genes and transcription factors mediate the fruit spine development in cucumber. Notably, despite many GO terms being commonly enriched in the upregulated genes in the *tbh* mutant and in spines of 1.6cm fruit (Fig. 6), only a few genes were shared in the two sets of upregulated genes (Fig. 4). Taken together with the fact that the *tbh* mutant has ‘tiny’ trichomes with a greatly reduced number of cells, and the spines of 1.6cm fruit have enormous base expansion with tremendously increased cell numbers compared with the spines of 0.5cm fruit, our data imply that distinct members of meristem regulators and transcription factors may regulate the *TBH*-mediated trichome development and spine base expansion in cucumber, and that some of them may function in opposite directions during cell division and trichome development.

**Table 2.** Examples of developmental regulators that were identified by DGE to be differentially expressed in the fruit spines of tbh mutant and WT

Gene ID	Gene name	Fold change	*P* value
**Meristem maintenance and regulation**			
Csa4G297540	*RPL* (*REPLUMLESS*)	3.6	5.0E–04
Csa3G144740	*ZLL* (*ZWILLE*)	2.0	1.9E–03
Csa1G064670	*ATML1* (*MERISTEM LAYER 1*)	3.2	1.6E–05
Csa6G497080	*BAM1* (*BARELY ANY MERISTEM 1*)	1.8	3.3E–03
Csa1G536820	*ARF6* (*AUXIN RESPONSE FACTOR 6*)	1.9	1.9E–03
Csa4G256430	*BOP2* (*BLADE ON PETIOLE2*)	2.7	2.9E–03
Csa3G736760	*AP2* (*APETALA 2*)	2.6	1.1E–04
Csa5G118190	*LPR1* (*Low Phosphate Root1*)	2.5	2.5E–04
**Sequence-specific DNA-binding transcription factor activity**			
Csa4G645830	*KNAT1* (*KNOTTED-LIKE FROM ARABIDOPSIS THALIANA*)	3.1	7.7E–05
Csa4G638510	*MYB77* (*myb domain protein 77*)	2.9	2.5E–07
Csa3G812750	*MYB105* (*myb domain protein 105*)	3.2	1.7E–04
Csa3G354520	*SPCH* (*SPEECHLESS*)	2.6	1.2E–04
Csa3G809420	*SPL9* (*SQUAMOSA PROMOTER BINDING PROTEIN-LIKE 9*)	2.2	4.7E–04
Csa2G369820	*ATHB22* (*HOMEOBOX PROTEIN 22*)	2.1	6.9E–04
Csa2G021550	*bHLH family protein* (*bHLH096*)	2.1	8.5E–04
Csa5G636510	*AtHB34* (*ARABIDOPSIS THALIANA HOMEOB-OX PROTEIN 34*)	2.1	3.3E–04

**Table 3.** Examples of developmental regulators that were identified by DGE to be differentially expressed in the spines of 1.6 and 0.5cm cucumber fruit

Gene ID	Gene name	Fold change (1.6/0.5cm)	*P* value
**Meristem maintenance**			
Csa7G041370	*STM* (*SHOOT MERISTEMLESS*)	37.8	3.4E–09
Csa1G408710	*ZLL* (*ZWILLE*)	9.0	1.8E–03
Csa1G025070	*PIN1* (*PIN-FORMED 1*)	19.9	1.7E–08
Csa6G139130	*LBD4* (*LOB DOMAIN-CONTAINING PROTEIN 4*)	6.3	4.6E–03
Csa4G103350	*DWF1* (*DWARF 1*)	3.7	1.2E–03
Csa5G608050	*NIK1* (*NSP-INTERACTING KINASE 1*)	9.2	4.6E–06
Csa6G109640	*SCL6* (*scarecrow-like transcription factor 6*)	3.5	1.8E–03
Csa5G642730	*NIK1* (*NSP-INTERACTING KINASE 1*)	6.6	1.9E–06
Csa5G092940	*BRL2* (*BRI1-LIKE 2*)	149.6	1.7E–06
**Sequence-specific DNA-binding transcription factor activity**			
Csa4G046650	*MNP* (*MONOPOLE*)	119.1	9.9E–06
Csa4G652000	*WRKY35* (*WRKY DNA-binding protein 40*)	53.5	4.3E–10
Csa1G505950	*Dof-type zinc finger domain-containing protein*	19.1	8.6E–09
Csa2G356610	*WOX4* (*WUSCHEL RELATED HOMEOBOX 4*)	157.2	1.0E–06
Csa3G895630	*ATHB-8* (*HOMEOBOX GENE 8*)	6.3	4.5E–07
Csa6G081510	*SHR* (*SHORT ROOT*)	214.4	1.5E–07
Csa6G135460	*APL* (*ALTERED PHLOEM DEVELOPMENT*)	214.4	4.6E–08
Csa1G423190	*AIL5* (*AINTEGUMENTA-LIKE 5*)	157.2	1.2E–06
Csa2G035350	*MYB50* (*MYB DOMAIN PROTEIN 50*)	286.7	6.2E–09
Csa6G526230	*WRKY35* (*WRKY DNA-binding protein 35*)	256.3	9.7E–08
Csa6G426940	*YAB2* (*YABBY2*)	3.7	6.7E–04
**Vascular patterning and polarity specification**			
Csa6G141360	*REV* (*REVOLUTA*)	4.7	1.4E–05
Csa6G525430	*PHB* (*PHABULOSA*)	3.4	9.2E–04
Csa3G194380	*KAN1* (*KANADI1*)	3.0	1.8E–03
Csa4G644740	*ANT* (*AINTEGUMENTA*)	8.2	4.5E–06
Csa6G496390	*ANT* (*AINTEGUMENTA*)	4.0	1.2E–04
Csa3G009500	*TRN2* (*TORNADO 2*)	17.7	3.2E–06

**Fig. 7.**
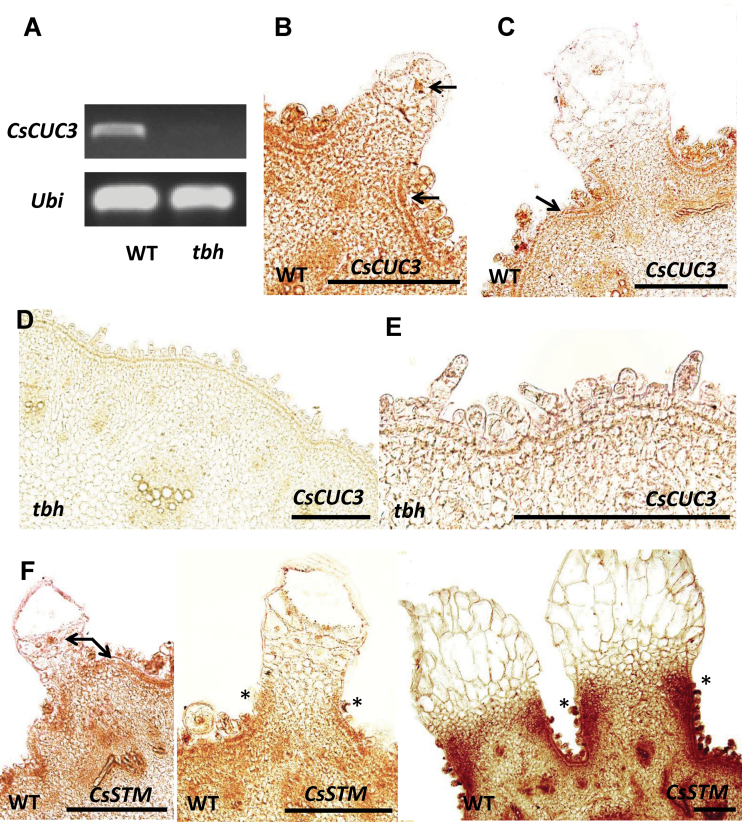
Expression analyses of *CsCUC3* and *CsSTM* during fruit spine development in cucumber. (A) Semi-quantitative RT-PCR of *CsCUC3* in pericarps of WT and *tbh* mutant. (B–F) Gene expression patterns as detected by *in situ* hybridization. *CsCUC3* was expressed in the epidermis and nucleus of spine cells in developing fruit spines of WT (arrows in B and C), but was undetectable in the *tbh* mutant (D, E). (F) Transcript accumulation of *CsSTM* was localized in the epidermis and nucleus of spine cells (arrows), and was strongly induced at the junction of the fruit base and wart during fruit base expansion (asteroids) in WT cucumber. Bars, 200 µm. (This figure is available in colour at *JXB* online.)

To explore further the role of meristem genes in trichome development, we examined the expression patterns of two meristem regulators, *CUP-SHAPED COTYLEDON3* (*CUC3*) and *STM* by *in situ* hybridization (Fig. 7). *CUC3* encodes a member of the NAC domain transcription factor family that is expressed in the meristem–organ boundaries and has been shown to regulate meristem organization, organ separation, and branching in *Arabidopsis* (Vroemen *et al.*, 2003; Hibara *et al.*, 2006). *STM*, which is expressed throughout the meristem but is downregulated in the organ primordia, encodes a member of the class I KNOX homeodomain transcription factors, which functions as a key regulator for meristem formation and maintenance (Clark *et al.*, 1996; Long *et al.*, 1996). In our DGE data, the expression of cucumber *CUC3* (*CsCUC3*) was over 1638-fold decreased in the *tbh* mutant, and we confirmed this downregulation via qRT-PCR (481-fold reduction) and semi-quantitative RT-PCR (Table 1, Fig. 7A). *In situ* hybridization showed that *CsCUC3* was strongly expressed in the epidermis of WT cucumber fruit, as well as in the nucleus of spine cells (arrows in Fig. 7B, C) but was almost abolished in the *tbh* mutant (Fig. 7D, E). This was consistent with the dramatic reduction in *CsCUC3* expression in the *tbh* mutant revealed by DGE and RT-PCR data, suggesting that the activation of *CsCUC3* by the TBH-related pathway may be important for fruit spine development in cucumber. Another meristem regulator, STM, was shown to be induced by 37.8-fold as detected by DGE and 22.8-fold as detected by qRT-PCR in the spines of 1.6cm fruit versus 0.5cm fruit (Table 1). Consistently, *in situ* hybridization showed that, in young spines before base expansion (on 0.5cm fruit), cucumber *STM* (*CsSTM*) was present in the epidermis and nucleus of spine cells (arrows in Fig. 7F, left). During spine development, *CsSTM* was strongly expressed in the junction between the spine base and fruit wart (denoted by asterisks in Fig. 7F, middle and right). This pattern is similar to the high accumulation of STM at the leaflet initiation sites in complex leaves (Bharathan *et al.*, 2002; Hake *et al.*, 2004), implying that STM may promote fruit base expansion in cucumber.

### Vascular patterning and polarity genes were induced during base expansion of cucumber fruit spines

Among the genes that were upregulated in the spines of 1.6cm fruit compared with those of 0.5cm fruit, genes that belong to GO terms ‘xylem and phloem pattern formation’ (*P*=1.0E–09) and ‘polarity specification of adaxial/abaxial axis’ (*P* =7.0E–09) were significantly enriched (Fig. 6B). Consequently, many well-known regulators for polarity specification and vascular patterning were significantly upregulated during fruit base expansion (Table 3). For example, the expression of cucumber homologues of the adaxial/xylem development marker *REVOLUTA* (*REV*) and the abaxial identity gene *KANADI1* (*KAN1*) was 4.7- and 3.0-fold higher, respectively, in the spines of 1.6cm fruit (Table 3) (Emery *et al.*, 2003; Juarez *et al.*, 2004; Nakata and Okada, 2012). *In situ* hybridization showed that *CsKAN* was expressed predominately in the nucleus of spine cells as well as in the epidermis (arrows in Supplementary Fig. S4 available at *JXB* online). Due to the presence of huge central vacuoles and squeezed internal organelles in the mature fruit spine, we were unable to locate the nucleus in the sections and were thus unable to detect a polarity pattern of *CsKAN* expression using *in situ* hybridization technology. However, we did qRT-PCR analyses and confirmed that *CsKAN* was indeed upregulated (4.5-fold) during fruit base expansion (Table 1), suggesting that polarity regulators may be involved in the fruit spine development in cucumber.

## Discussion

### Distinct morphology and developmental process of cucumber trichomes

Trichomes are the epidermal appendages that are mainly distributed on leaves, stems, and sepals (Hülskamp *et al.*, 1999; Hülskamp, 2004). Here, we found that cucumber trichomes not only cover the above-mentioned aerial organs but are also scattered on the surface of fruit (Fig. 1 and Supplementary Fig. S1 available at *JXB* online). Whether spines are present on cucumber fruit affects consumer preference and is thus of great economic importance. For example, in some European regions, people prefer glabrous cucumber without spines, whereas Asia customers desire cucumber with spines and warts. Cucumber trichomes belong to the multicellular non-glandular type of trichome. Each trichome consists of a stalk with four to eight cells lining up in a row and a dome-shaped base (Fig. 1G, H). Non-glandular trichomes have been shown to function in defence against insect herbivores, in protection against UV light and low temperature, and in facilitating seed dispersal in other plants (Hülskamp *et al.*, 1999; Hülskamp, 2004; Serna and Martin, 2006), and whether the non-glandular cucumber trichomes have the same functions warrants further studies.

Taking advantage of the large trichome base on cucumber fruit, we were able to explore the internal structure of trichomes using TEM. Fruit trichome cells display an enlarged cell size with a huge central vacuole and malformed internal organelles (Fig. 2A–D). Notably, abnormal-coloured plastids are often observed in the fruit spine cells (Fig. 2D and Supplementary Fig. S1A available at *JXB* online) but are very rare in epidermal cells, suggesting that cucumber trichomes may have a distinct origin from the epidermal-derived *Arabidopsis* trichomes (Marks, 1997; Hülskamp *et al.*, 1999; Larkin *et al.*, 2003). In addition, unlike the four rounds of endoreduplications in *Arabidopsis* trichome cells (32C) (Hülskamp *et al.*, 1994), cucumber trichome cells did not appear to undergo any endoreduplication (Fig. 2E, F). Consistently, cell-cycle-related genes were not differentially expressed in either of the DGE comparisons (*tbh* mutant vs WT, and 1.6 vs 0.5cm long fruit), supporting the suggestion that uni- and multicellular trichomes may be under different regulatory controls.

Compared with the complex developmental process of *Arabidopsis* trichomes (Szymanski *et al.*, 1998), formation of cucumber trichomes is relatively simple and can be generally divided into two steps: (i) initiation of the stalk and the base; and (ii) expansion of the base (Fig. 3). More importantly, the developmental phase of cucumber spines on each fruit surface is generally homogeneous, which makes it possible to isolate trichome populations in each specific developmental stage. Because cucumber is a horticultural crop of worldwide importance, and fruit spines directly affect the appearance and quality of cucumber fruit, detailed characterization of cucumber fruit trichomes will not only help understand the underlying molecular mechanisms of the development of multicellular non-glandular trichomes but will also pave the way for creating new cucumber varieties with desired trichome growth and density through breeding and genetic engineering. Therefore, cucumber fruit trichomes may serve as a model system for studying the development of multicellular non-glandular trichomes.

### *TBH* may function as a major activator during fruit spine development

Mutant analyses have identified both positive and negative regulators of *Arabidopsis* trichome development (Schwab *et al.*, 2000; Szymanski *et al.*, 2000). Although different cucumber varieties display enormous divergence in fruit trichome formation, no trichome mutant with developmental defects has been characterized yet. Here, we obtained and purified a spontaneous mutation *tbh*, and found that the morphology and development of trichomes was dramatically changed in the *tbh* mutant (Fig. 1). Disruption of the *TBH* locus led to tiny trichomes with a greatly reduced number of cells, aberrant cell shapes and organization, and branched trichomes (Fig. 1), suggesting that *TBH* may be required for cell division and directional growth of cucumber trichomes. In addition, transcriptome analyses by DGE showed that the downregulated genes were greatly outnumbered by the upregulated genes in the *tbh* mutant (Fig. 4, Supplementary Table S2 available at *JXB* online), implying that *TBH* may act as a primary activator during fruit spine development in cucumber. Considering that trichomes initiate but arrest their further development in the *tbh* mutant (Fig. 1), and that there are more trichomes in the *tbh* mutant than in WT, it is possible that TBH regulates both the initiation and outgrowth of trichomes in cucumber. Based on the activator–inhibitor model, initiated trichome cells may activate factors that inhibit trichome development in neighbouring cells (Hülskamp and Schnittger, 1998; Hülskamp, 2004). Therefore, TBH may act immediately after trichome initiation and its function is required for later development of trichomes as well as the activation of some inhibiting factor(s). Disruption of TBH function leads to arrested trichome growth and reduced inhibition for surrounding cells, which causes increased trichome density.

### New roles of meristem genes during fruit trichome development

The shoot apical meristem (SAM) is the ultimate origin for all above-ground parts of the plant body. It produces lateral organ primordia from the peripheral zone (PZ) while maintaining a pool of stem cells in the central zone (CZ) (Fletcher, 2002). The meristem regulator STM promotes the indeterminate meristematic cell fate in the SAM by limiting expression of *ASYMMETRIC LEAVES 1 (AS1)* and *AS2* in primordia. Both AS1 and AS2 inhibit the expression of KNOX family genes, which include STM, in the initiating primordia so as to stimulate cell differentiation and patterning (Byrne *et al*., 2000, 2002; Ori *et al.*, 2000). Overexpression of *STM* leads to switching the cell fate from determinate to indeterminate meristematic in leaf tissues (Smith *et al.*, 1992; Sinha *et al.*, 1993; Long *et al.*, 1996). Here, we found that ‘meristem maintenance’ genes were significantly induced in the *tbh* pericarps (epidermis plus spines) compared with in the WT (Fig. 6, Table 2). Therefore, the tiny trichomes with arrested development in the *tbh* mutant may be caused by the upregulated expression of meristem genes, which enables the trichomes to recapitulate a shoot meristem programme. Similarly, meristem maintenance genes were induced in the 1.6cm fruit compared with the 0.5cm fruit (Fig. 6, Table 3). However, only very few genes were commonly induced in both sets of comparisons (Fig. 4). Given that the CZ of SAM displays a greatly reduced number of cell divisions whereas the PZ cells divide much more frequently (Fletcher, 2002; Reddy and Meyerowitz, 2005), together with the fact that the *tbh* mutant had a reduced number of trichome cells with inert cell division, and that spines of 1.6cm fruit undergo rapid base expansion with active cell division, it is plausible that distinct members of meristem genes were upregulated in the *tbh* mutant and during spine base expansion. While negative regulators of cell division were induced in the *tbh* mutant, similar to those functioning in the CZ, positive regulators for cell division were promoted in the spines of 1.6cm fruit, similar to those acting in the PZ. In addition, it has been shown that the meristem–organ boundary gene *CUC3* promotes adventitious shoot formation and cell division in *Arabidopsis* (Daimon *et al.*, 2003). Here we found that transcripts of *CsCUC3* were abolished in the *tbh* mutant (Table 1, Fig. 7). However, neither the genomic sequence nor the 2.6kb promoter region of *CsCUC3* showed any difference between WT and the *thb* mutant (data not shown), implying that TBH regulates the expression of *CsCUC3* epigenetically or is an upstream regulator that is essential for *CsCUC3* expression and therefore cell division. During the expansion of the spine base, the meristem marker *CsSTM* was induced over 22-fold as detected by DGE and qRT-PCR (Table 1), and *in situ* hybridization showed that *CsSTM* was highly accumulated in the junction between the spine base and fruit wart (Fig. 7F). Previous studies have indicated that overexpression of *STM* leads to ectopic cell divisions (knots) in leaf tissue (Smith *et al.*, 1992; Sinha *et al.*, 1993), and that STM was induced at the leaflet initiation sites in complex leaves (Bharathan *et al.*, 2002; Hake *et al.*, 2004). These data suggested that, similar to the leaflet initiation, spine base expansion is mediated by *CsSTM* activation in cucumber. Interestingly, we did not detect any differential expression of the homologues of well-characterized trichome regulators such as *TTG1* (Galway *et al.*, 1994; Walker *et al.*, 1999), *GL1*, *GL2*, *GL3*, and *EGL3* (Payne *et al.*, 2000; Zhang *et al.*, 2003), *TRY* (Schellmann *et al.*, 2002; Pesch and Hülskamp, 2011), and *CPC* (Wada *et al.*, 1997, 2002) in either set of DGE data. One possibility is that most of these genes act in the trichome initiation or shortly after initiation, and our samples for the DGE analyses were collected at a time point that was too late to detect any changes in their expression. Another possibility is that the unicellular *Arabidopsis* trichomes and multicellular cucumber spines may be under the control of distinct transcription networks. Consistent with this notion, unlike the common regulatory mechanism for trichome and root hair development in *Arabidopsis* (Hülskamp, 2004; Pesch and Hülskamp, 2004), neither meristem genes nor known trichome regulators showed significantly differences in the roots of *tbh* mutant and WT (Supplementary Table S4 available at *JXB* online). Future studies using reverse genetics strategies will help uncover the regulatory roles of meristem genes and known trichome regulators during cucumber fruit spine development.

### Conserved requirement of polarity regulators for organ expansion

Polarity specification of the adaxial–abaxial axis has been well characterized in leaf patterning (Yamaguchi *et al.*, 2012). Generally, the vascular bundles are also polarized, with the xylem being adaxial and the phloem being abaxial. Previous studies have identified adaxial-specific genes such as *PHABULOSA*, *PHAVOLUTA*, and *REVOLUTA* (*REV*) (Eshed *et al.*, 2001; McConnell *et al.*, 2001; Otsuga *et al.*, 2001), and abaxial-specific regulators such as *FILAMENTOUS FLOWER* and *KANADI* (Sawa *et al.*, 1999; Emery *et al.*, 2003; Juarez *et al.*, 2004; Nakata and Okada, 2012). A model has been proposed in which juxtaposition of adaxial and abaxial developmental fields is required for laminar expansion and margin development in leaves (Eshed *et al.*, 2001; Emery *et al.*, 2003; Nakata and Okada, 2012). Disruption of either adaxial- or abaxial-specific genes causes a partial or completely filamentous leaf phenotype that is defective in laminar expansion (Sawa *et al.*, 1999; McConnell *et al.*, 2001; Otsuga *et al.*, 2001; Emery *et al.*, 2003). In this study, we found that GO terms ‘xylem and phloem pattern formation’ and ‘polarity specification of adaxial/abaxial axis’ were significantly enriched among the upregulated genes in the spines of 1.6cm fruit (Fig. 5B, Table 3), and we verified that both adaxial and abaxial identity genes were promoted during spine base expansion (Tables 1 and 3, Fig. 6). These data suggested that, similar to the laminar expansion in leaf development, both adaxial and abaxial regulators are required for cucumber spine base expansion. It would be interesting to explore whether auxin mediates the adaxial and abaxial juxtaposition in cucumber trichome development as in the leaves (Wang *et al.*, 2011), and what are the expression patterns of the above-mentioned polarity markers in the mature dome-shaped spine base of cucumber fruit in future studies.

## Supplementary data

Supplementary data are available at *JXB* online.

Supplementary Fig. S1. Trichome distribution in WT cucumber and the *tbh* mutant.

Supplementary Fig. S2. Two types of trichomes in cucumber as observed by scanning electron microscopy.

Supplementary Fig. S3. Developmental stages of cucumber fruit spines.

Supplementary Fig. S4. Gene ontology (GO) terms that were significantly enriched (*P*<0.01) in the downregulated genes in the *tbh* mutant or in the spines of 1.6cm long fruit.

Supplementary Table S1. Summary of digital gene expression sequencing data.

Supplementary Table S2. List of genes that are differentially expressed in fruit spines of the WT and *tbh* mutant.

Supplementary Table S3. List of genes that are differentially expressed in fruit spines of two different developmental stages.

Supplementary Table S4. qRT-PCR analysis of meristem genes and trichome regulators in the leaf and root of *tbh* mutant versus those in WT cucumber.

Supplementary Table S5. List of primers used in this study.

Supplementary Data

## Abbreviations:

bHLH: basic helix–loop–helix
CZ: central zone
DAPI: 4’,6-diamidino-2-phenylindole
DGE: digital gene expression
FDR: false discovery rate
GO: gene ontology
PZ: peripheral zone
qRT-PCR: quantitative reverse transcription-PCR
SAM: shoot apical meristem
SEM: scanning electron microscopy
TEM: transmission electron micrograph
WT: wild type.
