# Supplementary material for: Transcriptome profiling reveals roles of meristem regulators and polarity genes during fruit trichome development in cucumber (Cucumis sativus L.)
Source: J Exp Bot. 2014 Jun 24;65(17):4943–58. doi: 10.1093/jxb/eru258 (PMC4144775; doi:10.1093/jxb/eru258)
Supplement: Supplementary Data [file supp_65_17_4943__index.html]

Transcriptome profiling reveals roles of meristem regulators and polarity genes during fruit trichome development in cucumber (Cucumis sativus L.) — Transcriptome profiling reveals roles of meristem regulators and polarity genes during fruit trichome development in cucumber (Cucumis sativus L.) — Supplementary Data 

# Transcriptome profiling reveals roles of meristem regulators and polarity genes during fruit trichome development in cucumber (*Cucumis sativus* L.)

## Supplementary Data

Data files

**Files in this Data Supplement:**

- Supplementary Data - Supplementary Data
- Supplementary Data - Supplementary Data
- Supplementary Data - Supplementary Data
