# Supplementary material for: Transcriptome profiling reveals roles of meristem regulators and polarity genes during fruit trichome development in cucumber (Cucumis sativus L.)
Source: J Exp Bot. 2014 Jun 24;65(17):4943–58. doi: 10.1093/jxb/eru258 (PMC4144775; doi:10.1093/jxb/eru258)
Supplement: Supplementary Data [file supp_eru258_jexbot125823_file001.pdf]

# Figure S1

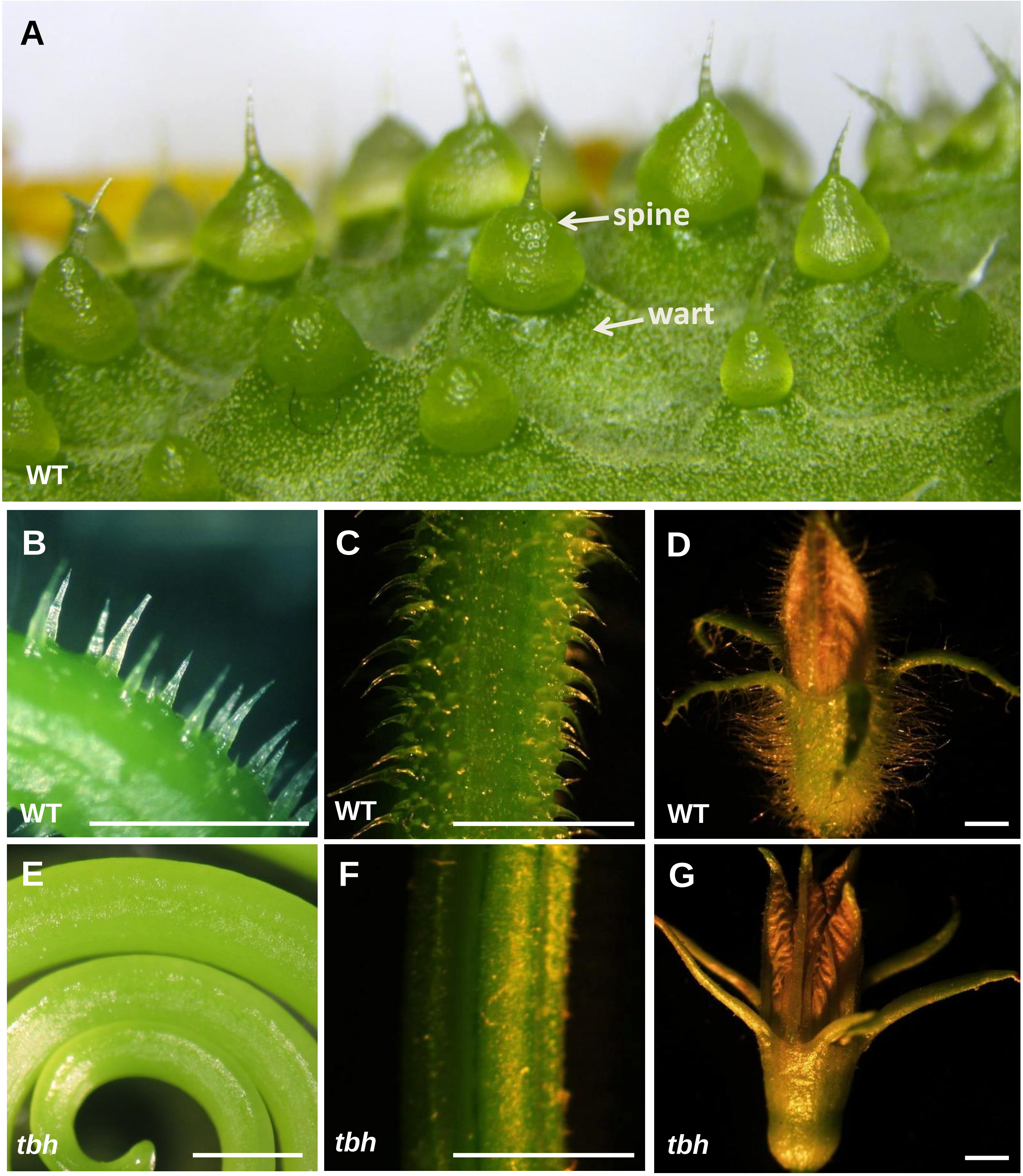

**Supplemental Figure S1. Trichome distribution in WT cucumber and *tbh* mutant.**

(A) Optical microscope of cucumber fruit surface showed the mature spine (light green) on the top and developing wart on the bottom. (B-G) Trichomes were evenly scattered on the surface of tendrils (B, E), stems (C, F), and male flower buds (D, G) in WT cucumber (B-D), but were barely visible in the *tbh* mutant (E-G) under optical microscope. Scale bars represent 3mm.

## Figure S2

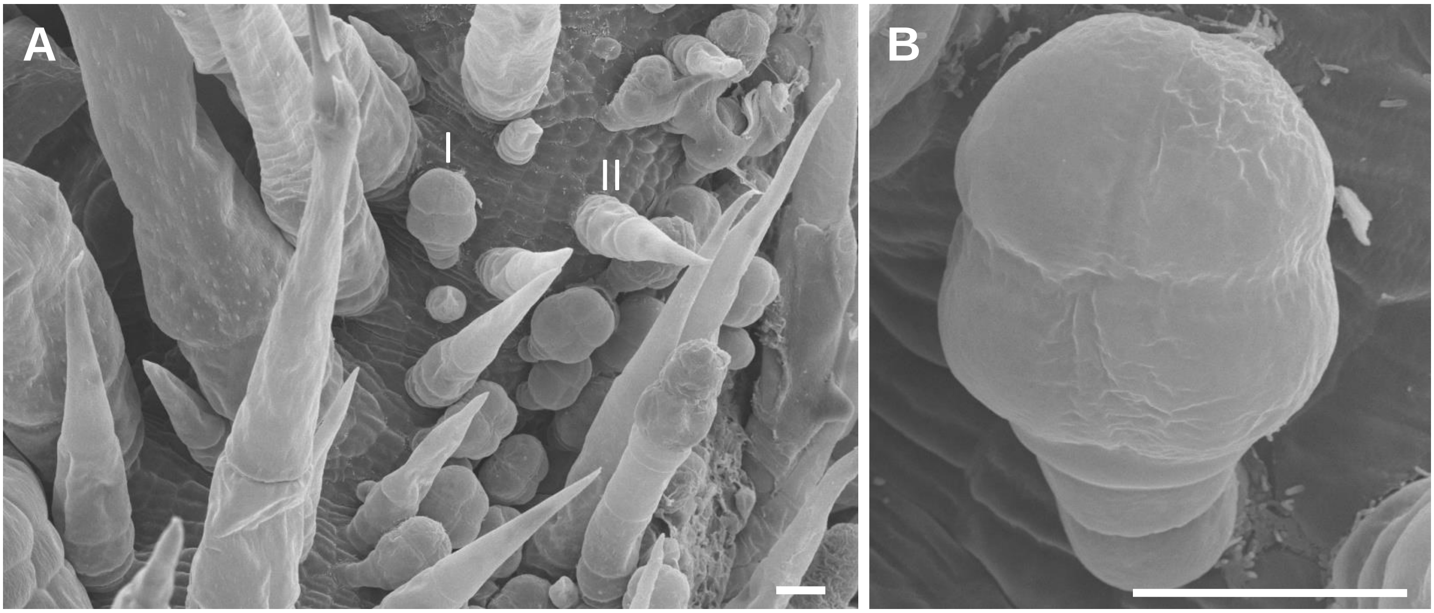


**Supplemental Figure S2. Two types of trichome in cucumber as observed by scanning electron microscopy**. (A) SEM of a young leaf showed the type I and type II trichomes in cucumber. (B) Enlarged view of a typical type I trichome in cucumber. Scale bars represent 20μm.

Figure S3

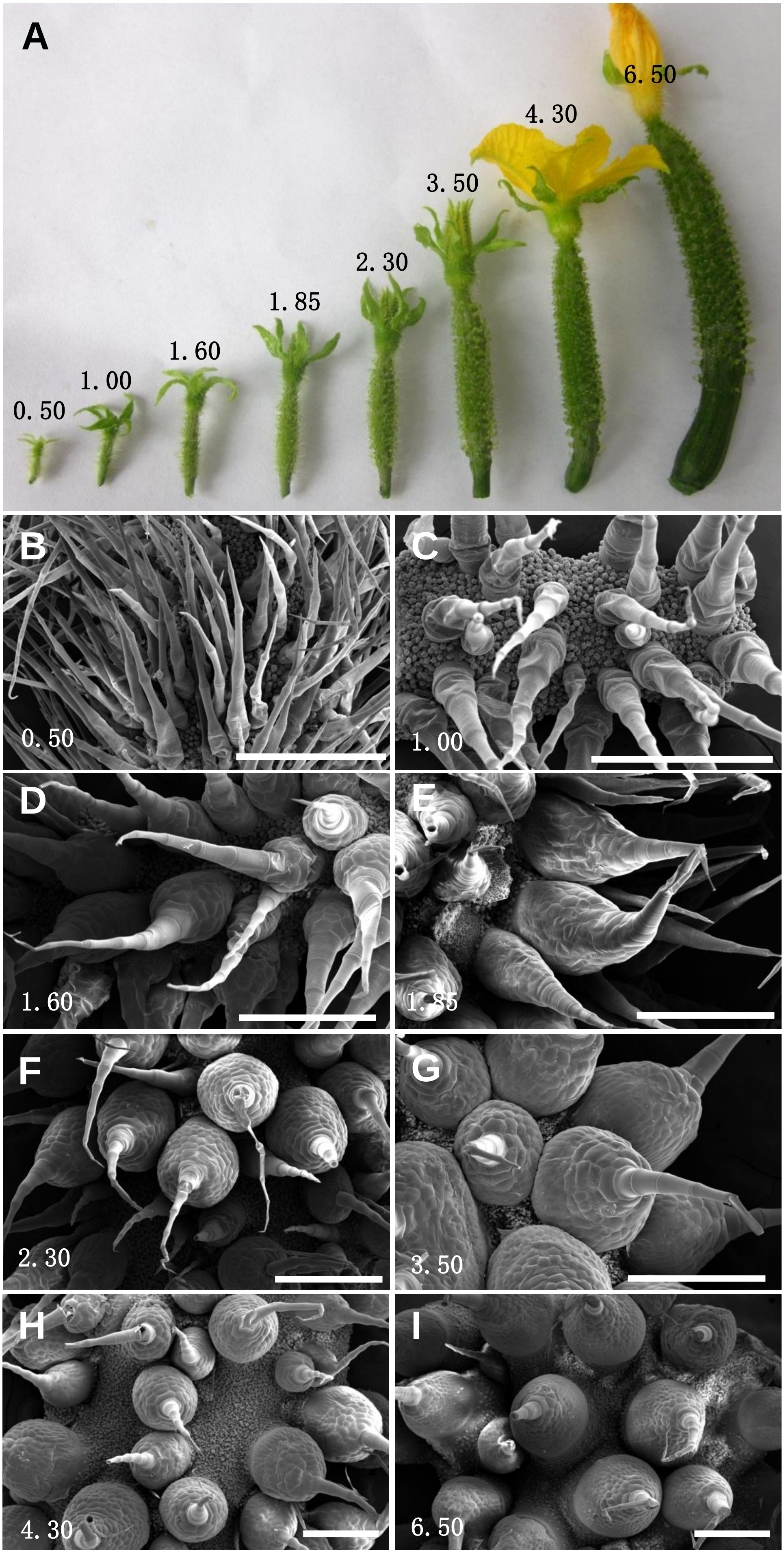
A
0. 50
1. 00
1. 60
1. 85
2. 30
3. 50
4. 30
6. 50
B
0. 50
C
1. 00
D
1. 60
E
F
2. 30
G
3. 50
H
4. 30
I
6. 50

**Supplemental Figure S3. Developmental stages of cucumber fruit spines.**

(A) Eight developmental stages we used to observe the morphology of fruit spines. (B-I) SEM of fruit spine development during above eight stages. The numbers indicated the average fruit lengths in cm.

# Fig S4

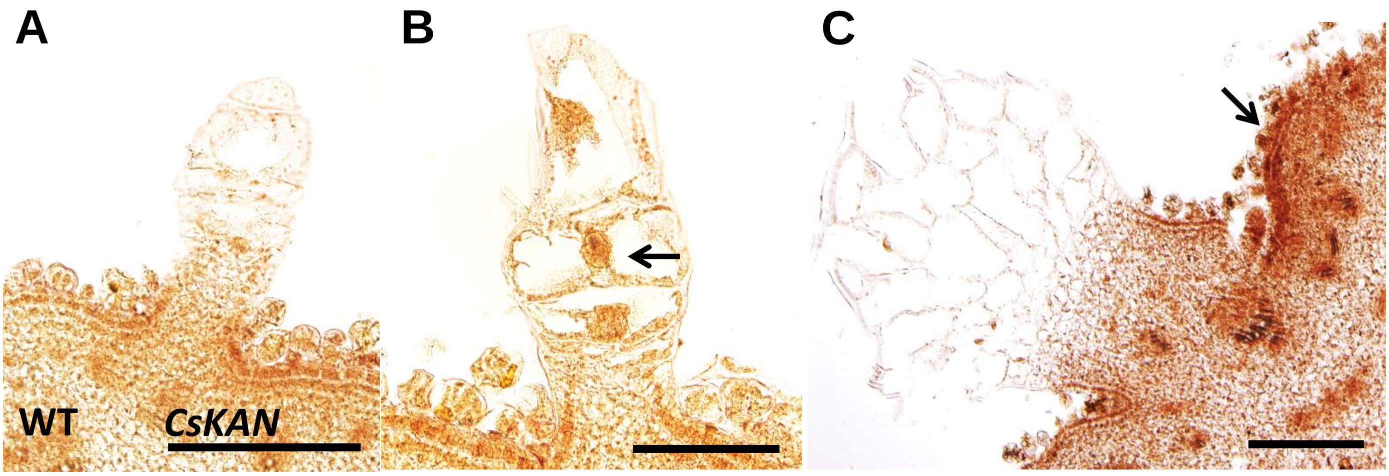


**Supplemental Figure S4. Expression analyses of *CsKAN* during fruit spine development in cucumber**.
(A-C) Distribution of *CsKAN* in developing fruit spines of WT cucumber. Arrows show the high expression of *CsKAN* in spine nucleus as well as in the fruit epidermis. Scale bars = 200 µm.

**Table S1. Summary of digital gene expression sequencing data**

| | WT vs. *tbh* | | | | 1.6cm vs. 0.5cm | | | |
|---|---|---|---|---|---|---|---|---|
| | **WT_rep1** | **WT_rep2** | **tbh_rep1** | **tbh_rep2** | **1.6cm_rep1** | **1.6cm_rep2** | **0.5cm_rep1** | **0.5cm_rep2** |
| Number of raw reads | 7,137,255 | 7,490,771 | 6,953,507 | 7,063,193 | 7,164,599 | 7,067,024 | 7,088,068 | 7,494,811 |
| Number of clean tags | 6,956,882 | 7,308,968 | 6,795,946 | 6,892,017 | 6,989,699 | 6,894,716 | 6,921,677 | 7,335,259 |
| Percentage of clean tags (clean tags/ | 97% | 98% | 98% | 98% | 98% | 98% | 98% | 98% |
| Number of unique clean tags | 163,250 | 167,896 | 173,256 | 169,915 | 169,704 | 153,134 | 145,713 | 135,858 |
| Number of mapped tags | 6,635,229 | 7,008,964 | 6,456,738 | 6,556,836 | 6,638,619 | 6,384,792 | 6,570,588 | 6,920,718 |
| Percentage of mapped tags (mappe[illegible] | 95% | 96% | 95% | 95% | 95% | 93% | 95% | 94% |
| **Number of mapped unique tags** | 135,844 | 141,065 | 147,073 | 139,267 | 137,046 | 124,399 | 120,224 | 109,384 |

**Table S4. qRT-PCR analysis of meristem genes and trichome regulators in the leaf and root of *tbh* mutant versus those in WT cucumber**

| Gene ID | Putative Annotation | Leaf | Root |
|---|---|---|---|
| Csa4G297540 | RPL (REPLUMLESS) | -1.7±0.06 | -1.1±0.24 |
| Csa3G736760 | AP2 (APETALA 2) | -1.5±0.32 | 1.0±0.08 |
| Csa7G041370 | STM (SHOOT MERISTEMLESS) | 1.2±0.21 | 1.2±0.36 |
| Csa5G608050 | NIK1 (NSP-INTERACTING KINASE 1) | -2.1±0.49 | 1.1±0.06 |
| Csa5G092940 | BRL2 (BRI1-LIKE 2) | -1.8±0.25 | -1.0±0.29 |
| Csa4G256430 | BOP2 (BLADE ON PETIOLE2) | 4.5±0.09 | -1.4±0.18 |
| Csa3G144740 | ZLL (ZWILLE) | -2.5±0.06 | -1.6±0.20 |
| Csa6G497080 | BAM1 (BARELY ANY MERISTEM 1) | -3.0±0.31 | 1.5±0.06 |
| Csa4G097650 | TTG1 (TRANSPARENT TESTA GLABRA 1) | 1.6±0.17 | -1.1±0.08 |
| Csa7G447000 | GL2 (GLABRA 2) | -1.9±0.23 | -1.7±0.08 |
| Csa3G824850 | MYB106 (myb domain protein 106) | -1.8±0.19 | 1.8±0.12 |
| Csa4G052630 | ADL1 (ARABIDOPSIS DYNAMIN-LIKE PROTEIN) | -1.7±0.45 | -1.4±0.27 |
| Csa6G003480 | GL3 (GLABRA3) | -11.3±0.08 | -1.3±0.40 |
| Csa5G139610 | TRY(TRIPTYCHON) | 2.7±0.02 | -1.4±0.47 |

## Table S5. Primer information used in this study

| **Primers for qRT-PCR** | |
|---|---|
| *ADL1-F* | 5'- TTTCCTTGGGTTGGTGTTGTG-3' |
| *ADL1-R* | 5'- ATCCTATGGGCGAGGTGCTT-3' |
| *ANT-F* | 5'- AGACAAGTTTATCTCGGTGGCTAT-3' |
| *ANT-R* | 5'- TAAATGTGTAGAGGAACCCCAG-3' |
| *AP1-F* | 5'- AAAGAGAAGAGTGTAGCATTGGC-3' |
| *AP1-R* | 5'- ATGTTTAGAGAGGGATGAGGAGG-3' |
| *AP2-F* | 5'- CGACGAACAGGAAGATGGGA-3' |
| *AP2-R* | 5'- GCTTCCCCTTGAAAATCCAGTA-3' |
| *ATHB8-F* | 5'- CAATGCTGTGCTTTGTGCCA-3' |
| *ATHB8-R* | 5'- ATCAATACTGCTGTCTGCCC -3' |
| *ATML1-F* | 5'- AGGGGAGGAAGAGCGTTATG -3' |
| *ATML1-R* | 5'- CCACTGATTGCGGGTGTTT -3' |
| *BAM1-F* | 5'- CCCTCAAAACCTCCATTACCG -3' |
| *BAM1-R* | 5'- GCAAGTGACCCCAAACCAAGT -3' |
| *BMY7-F* | 5'- CATTCATCTCACTCCAGAGTACCGAC -3' |
| *BMY7-R* | 5'- CCAACGCCATCATCACAAACAC -3' |
| *BOP2-F* | 5'- CACCACGCTGATCCAACCAT -3'-3' |
| *BOP2-R* | 5'- ATCCCTGGCACTGCTCCTTT -3' |
| *BRL2-F* | 5'-GGGAAACGACCAACCGATAA-3' |
| *BRL2-R* | 5'-CTCTCGCAGCATTGTCACCA-3' |
| *CUC3-F* | 5'- CACCATAGATTATTGCCACCAGG -3' |
| *CUC3-R* | 5'- GAAGTTCCCACGGCTCACAT -3' |
| *GA5-F* | 5'- CATTACCTTCGCACCAAACTCG -3' |
| *GA5-R* | 5'- CCATTATCCCCAAAGATAGTCCACTC -3' |
| *GL2-F* | 5'- TCAACGGTTGTCTATGCTCAAG -3' |
| *GL2-R* | 5'- GAACCCTGTCGGTAATGTCGT -3' |
| *GL3-F* | 5'- GATTTTCTGGTCACCGTCGTC -3' |
| *GL3-R* | 5'- GCTTGAACTGTTTTCCTCGTCTTG -3' |
| *HB-5-F* | 5'- TGGCTTCTGAATTGGGACTTG -3' |
| *HB-5-R* | 5'- CAGCTTCTTGTTCTTCCACCGT -3' |
| *KAN-F* | 5'- CAATACACCAACTTTGTCAACAC -3' |
| *KAN-R* | 5'- TCTGCTCTGCCTAATCTGAACTC -3' |
| *KNAT6-F* | 5'- CTCACACACGCACTGTCTCTT -3' |
| *KNAT6-R* | 5'- GACCTTTGTCTTCTAACCTCA -3' |
| *LBD2-F* | 5'- CGTCTCGCTCGTTACGCTAA -3' |
| *LBD2-R* | 5'- TGGATAAGGAAAATGGGTGAAAT -3' |
| *LRP1-F* | 5'- GTGGGAACCAAGCCAAGAAA -3' |
| *LRP1-R* | 5'- CGTCTGTGAAGCAATCAACCTC -3' |
| *MYB106-F* | 5'- GCCGGTCTTGAGAGATGTGG -3' |
| *MYB106-R* | 5'- TTGCTATGGCAGACCACCTG -3' |
| *NIK1-F* | 5'-TTGATGATTATTGCGAGGCTGT-3' |
| *NIK1-R* | 5'-GTTTTCTCGGAAGATTGACCAG-3' |
| *PDF2-F* | 5'- CATTGAAAATTCCCGCTTGC -3' |
| *PDF2-R* | 5'- TGGACCGAAGTTAGTGATGCC -3' |
| *RPL-F* | 5'-ATTCCGCCGTTTTTGACTCC-3' |
| *RPL-R* | 5'-ATGGCTTTTATGGCTAAGTTTGC-3' |
| *SHY-F* | 5'- CCGCTACTGCCACAGGAAAA -3' |
| *SHY-R* | 5'- CATAAGGGGAAGCCAACTCA -3' |
| *SHY2-F* | 5'- TTTCAAAATGGGTTAAACCTCG -3' |
| *SHY2-R* | 5'- GTTGATTCTGATGGATTGAGGT -3' |
| *STM-F* | 5'- TATCCCTCGGAATCGCAAAAG -3' |
| *STM-R* | 5'- ACCAGCATCCATCACCACAAA -3' |
| *TRY-F* | 5'- ACAATCATCGTCACCAGAAACC -3' |

| | |
|---|---|
| *TRY-R* | 5'- ATCCCACCTATCTCCAATCAGC -3' |
| *TTG1-F* | 5'- CTCCTCAACAACAGCAAAACCA -3' |
| *TTG1-R* | 5'- CCCCAAGCAATGTCATAAACC -3' |
| *YAB2-F* | 5'- AATGCTGGGGATTCAAGTAAGGA -3' |
| *YAB2-R* | 5'- AGGAGGGATTCTATGTTGGTCAC -3' |
| *ZLL-F* | 5'- GCTTGTGGATGAAGAAGATGGAG-3' |
| *ZLL-R* | 5'- CCTTTTGGATGAAAGCTCTCTCA-3' |
| *UBI-R* | 5'- CACCAAGCCCAAGAAGATC -3' |
| *UBI-F* | 5'- TAAACCTAATCACCACCAGC -3' |
| ***Primers for cloing*** | |
| pCUC3-F | ACTTCGATTAGATCCAAGACAGAA |
| pCUC3-R | TAACCTGGTGGCAATAATCTATGG |
| gCUC3-F | ATGTTTGGAATTGAAGAAGTTATTGGA |
| gCUC3-R | TCAGCACCCTCCGGACCAA |
| **Primers for *in situ* probes** | |
| *CUC3-SP6* | 5'- GATTTAGGTGACACTATAGAATGCT CACCATAGATTATTGCCACCAGG -3' |
| *CUC3-T7* | 5'- TGTAATACGACTCACTATAGGG GAAGTTCCCACGGCTCACAT -3' |
| *KAN-SP6* | 5'- GATTTAGGTGACACTATAGAATGCT CAATACACCAACTTTGTCAACAC -3' |
| *KAN-T7* | 5'- TGTAATACGACTCACTATAGGG TCTGCTCTGCCTAATCTGAACTC -3' |
| *STM-SP6* | 5'- GATTTAGGTGACACTATAGAATGCT TATCCCTCGGAATCGCAAAAG -3' |
| *STM-T7* | 5'- TGTAATACGACTCACTATAGGG ACCAGCATCCATCACCACAAA -3' |
